# Alcohol Use among High School Learners in the Peri-Urban Areas, South Africa: A Descriptive Study on Accessibility, Motivations and Effects

**DOI:** 10.3390/children9091342

**Published:** 2022-09-01

**Authors:** Mmampedi Mathibe, Lindiwe Cele, Perpetua Modjadji

**Affiliations:** 1 Department of Public Health, School of Health Care Sciences, Sefako Makgatho Health Sciences University, 1 Molotlegi Street, Ga-Rankuwa 0208, South Africa; 2 Non Communicable Diseases Research Unit, South African Medical Research Council, Cape Town 7505, South Africa

**Keywords:** alcohol use, accessibility, motivations, effects, high school learners, peri-urban, Tshwane, South Africa

## Abstract

Learners are vulnerable to alcohol use and its negative effects, largely due to accessibility of alcohol products, especially in the localities with poor socioeconomic status and infrastructure. This study aimed to determine the accessibility, motivations and effects of alcohol use among high school learners (n = 403) in Tshwane North and West, South Africa, using a descriptive, cross-sectional design. Data were collected using a validated questionnaire and analysed using STATA 17. Learners (16 ± 2 years) had poor demographic status and lifestyle behaviors. Availability of alcohol outlets (54%) near schools was reported, and learners indicated easy access (65%) to taverns and bottle stores (30%), and purchasing alcohol without a proof of identity document (70%). Motivations for alcohol use were self-pleasure (36%), coping with stress (24%) and increasing self-esteem (19%). Almost half of the learners (49%) introduced themselves to alcohol use, while others were influenced by friends (36%) and family (14%). Reported alcohol related effects were a negative impact on health (56%), brain function (25%) and school work (12%), in addition to social harms, including problems with friends (25%) and parents (17%), physical fights (19%) and engaging in risky sexual behaviour (11%). Effective strategies are necessary to address underage alcohol use and should include regulating the proximity of alcohol outlets to schools, life skills training to address learners’ drinking motives and constantly alerting parents about the relevance of modeling behaviour.

## 1. Introduction

Alcohol use and abuse is increasingly becoming a worldwide lifestyle that is prevalent in rich and poor countries alike and one of the top three major health problems [1,2]. In sub-Saharan Africa (SSA), the prevalence of substance use among adolescents is approximated at 42%, with alcohol (40.8%) and tobacco (45.6%) being the highest prevailing substances used across Africa continent, compared to other substances [3,4]. In particular, South Africa has growing evidence of risky alcohol use among various population groups [5,6], in addition to tobacco use [7,8,9,10]. The country continues to experience considerable change with the onset age of alcohol intake among school age adolescents across the society [11,12,13,14]. Studies in South Africa have estimated youth drinking at 32%, especially between the ages of 11–20 years, while 12% of teenagers are reported to initiate alcohol use before 13 years of age [14,15,16]. Additionally, recent data showed the prevalence of alcohol use among high school learners ranging from 22% to 69% [17,18,19,20,21]. 

Accessibility and availability of alcohol from various sources increase alcohol use among adolescents, including direct purchasing from the commercial establishments, despite laws prohibiting such sales to young people under the age of 18 years [22], which is also the case in South Africa [23,24]. Literature suggests that alcohol availability is categorised in terms of physical, economic, social and subjective or psychological availability [25]. Physical availability refers to the arrangements made by governments that determine the convenience or difficulty to obtain and consume alcoholic beverages. Economic availability refers to the price and disposable income affecting the affordability of alcohol. Social availability refers to the degree of normative support for drinking provided by a person’s key social groups, such as family, friends, sports clubs and the neighbourhood public house. Subjective or psychological availability refers to the perception of accessible people on alcohol and their response to alcohol marketing [26]. Researchers in other countries, including South Africa, have reported an established positive link between the number of liquor outlets found in a locality, as well as proximity, density of alcohol outlets and indulgence in alcohol use in the communities [24,27,28,29,30,31]. 

A combination of different variables has been implicated to influence the use of alcohol among adolescents [32]. Adolescence is a developmental period of heightened responsiveness to social rewards, and this implies an increasing desire to fit in among peers [33]. Drinking motives and perceived peer and parental norms are strong predictors of alcohol use [34]. Identifying the adolescents’ motivations for their alcohol use has revealed that adolescents consume alcohol when they are having fun with their friends. Moreover, adolescents use alcohol to overcome the stressful life events and conflict situations in relationships with their family and friends, while for others it is self-reward after an achievement [35]. Further literature documents that alcohol use during adolescence is influenced by learning to use alcohol based on social observation of other significant persons’ behaviours (family members, friends, peers, social models), developing positive attitudes towards alcohol and its effects and a result of social imitation and social modelling processes by peer influences (i.e., social learning), as reported by Bențea [36]. Therefore, personal motivations and perceived social norms influence adolescent alcohol use [34]; it can be deduced that most adolescents consume alcohol as a result of the pleasure, peer group, and family influence [34,36]. In addition to South Africa [17,37], studies in other countries, such as Israel [38], Namibia [28,39], and Spain [36] have confirmed drinking motives and influences of significant persons among adolescents. 

Alcohol is an addictive, psychoactive substance that can cause significant harm to the individual if consumed excessively [40]. Alcohol use among adolescent is associated with many serious social and developmental issues and behaviour problems, such as school absenteeism, acts of aggression and violence, suicidal behaviour, delinquency, antisocial behaviours, neuropsychiatric disorders, etc. [36]. Alcohol use disorders in adolescence and later in adulthood are attributed to early initiation of alcohol use among school age adolescents [41,42]. As previously stated, substance use among adolescents can lead to an increased risk of transmission of sexually transmitted infections, juvenile delinquency and other problems associated with physical and mental health [43]. Specifically, alcohol misuse is associated with an increased number of sexual partners, unprotected sex and sexual encounters that are later regretted [44,45]. Morojele and colleagues [46] reported that adolescents who drink are more likely to have early sexual debut and engage in multiple sexual partnerships. 

Accessibility of alcohol products in the localities with poor socioeconomic status and infrastructure have been associated with increased alcohol use, coupled with drinking motives, manifesting related negative effects and social harms. Increase in alcohol outlets in the Southern African countries such as South Africa calls for concern as it has become a social and a political issue [47]. Our study considered problem drinking based on many studies reported on alcohol use among high school learners in South Africa [18,21,47]. However, there is limited research in the townships (i.e., per-urban) on the drivers of alcohol use among adolescents in terms of access to alcohol outlets, drinking motives and harmful effects, except for minimal research [17,19,20]. The current study will enable understanding the context in which alcohol is accessed by learners by exploring by concentrating on the accessibility, motivations and effects of alcohol use among high school learners in Tshwane North and West, South Africa.

## 2. Methods and Materials

### 2.1. Study Design and Frameworks

A descriptive cross-sectional study using a quantitative method was conducted among high school learners to determine to accessibility, motivations and effects of alcohol use in the context of poor demographics and infrastructure. In conducting this study, the ecological system theory was relevant to study alcohol issues among high school learners in relation to the external environmental factors, such as taverns and bottle stores. The proximity of alcohol outlets to schools predisposes learners to lifestyle behavioural change. The theory further explains how people living in these communities learn behaviours and grow through interaction, inevitably influencing one another in every aspect of life [48]. The application of the theory to the current study shows the influence of the external environment on individual behaviours. Therefore, interacting with alcohol outlets puts learners at more risk of consuming alcohol than those who are less exposed to alcohol outlets [49,50]. In addition, we also integrated the motivational model, postulating that individuals drink to obtain positive outcomes or to avoid negative consequences (i.e., valence), and they are motivated by internal or external rewards (i.e., source). There are four categories of motivations. The first one is drinking to enhance positive mood (i.e., enhancement), followed by drinking to obtain social rewards (i.e., social motive), then drinking to cope with negative emotions (i.e., coping) and finally, drinking to avoid social rejection (i.e., conformity) [51]. Previous research has found that drinking motives are strong predictors of alcohol consumption and are the most proximal antecedents of alcohol use [35].

### 2.2. Study Setting and Population

The study was conducted in the peri-urban settings situated in the City of Tshwane municipality, in Gauteng Province (i.e., Soshanguve and Winterveldt). Peri-urban areas, also called townships in South Africa, are underdeveloped residential areas reserved for mostly Africans to live, originating from the Apartheid era [52] and are mostly characterized with poor infrastructure and low socioeconomic status [17,53]. There are 22 high schools located in Soshanguve, with 16 high schools in Tshwane North and 6 high schools in Tshwane West, with a total enrolment of approximately 24,000. Schools in these localities depend on funding from the South African government (i.e., quintile three) [54]. Learners attending high schools in the abovementioned areas participated in the study. These learners received parental consent to be part of the study, gave individual written consent (learners aged ≥ 18 years) and assent (learners below 18 years old) (i.e., inclusion criteria). The study excluded learners without parental consent for participation. According to the literature, the adolescent population structure can be classified into two groups, which are the early adolescent population aged 10–14 years and the late adolescent population comprising those aged ≥ 15 years [55].

### 2.3. Sample Size and Sampling Techniques

A minimum representative sample size of 379 was calculated using a validated Raosoft^®^ online sample size calculator (Raosoft Inc., Seattle, WA, USA), considering a total population size of ±24,000 learners, a 95% confidence level and 50% response distribution. To cater for a non-response, the sample was buffered with 10% and increased to 416. After 406 adolescents responded (97% response rate), and 3 were discarded with missing data, a final sample size of 403 was obtained. A multistage sampling was used, firstly randomly selecting 3 high schools out of the 23 schools, followed by a random selection of learners from the lists of learners who have obtained parental consent. After obtaining the permission to conduct the study from the schools’ governing body, the main researcher visited the selected schools to recruit and engage learners on the procedure and preparations for data collection with the help of the nutrition teachers. All engagements were conducted during breaks and after schools, and classes were not interrupted.

### 2.4. Data Collection and Tool

Data were collected using a validated researcher-administered questionnaire, adapted from studies conducted among high school learners [17,19,56] and on motivations for alcohol use [34,36]. Data collected included the demographics, accessibility, motivations and effects of alcohol use among learners. The questionnaire was validated for content, representing all aspects of the constructs on accessibility, motivations and effects of alcohol use, in addition to face validity, which presented all variables to be measured at face value. Following the approval of experts on the suitability of the questionnaire, a mini-study was conducted among 30 learners who did not form part of the main study to pre-test the tool (i.e., questionnaire) for feasibility. During a pilot study, the research assistants who speak the local language (i.e., Setswana) were taken through the process of conducting preliminary interviews for training purposes by the main researcher. After pretesting the questionnaire, slight changes were implemented on wording and layout of the questionnaire.

### 2.5. Data Analysis

Data were analysed using STATA 17 (StataCorp. 2021. Stata Statistical Software: Release 17., StataCorp LLC, College Station, TX, USA). Missing data were identified through complete case analysis, and three questionnaires with ≥10% of missing data were discarded. Descriptive statistics in the form of frequencies (n) and percentages (%) were used to summarise all the categorical variables for accessibility, motivations and effects of alcohol use, and further analyses on comparison by groups were computed using Chi-square (ꭓ2) and Fishers’ exact for variables with expected values less than five in a cell. Significance level was set at *p* < 0.05.

### 2.6. Ethical Considerations

Ethical approval was obtained from the Sefako Makgatho Health Sciences University Research and Ethics Committee (SMUREC). The project was approved on 1 March 2018, with the projection identification quote of SMUREC/H/51/2018: PG. The study received permission from the Tshwane Department of Education (Ref 8/4/4/1/2). Written informed consent was obtained from parents of all learners and from learners aged 18 years and above, while leaners below 18 years gave assent. 

## 3. Results

### Demographic Characteristics of School Learners

The demographics of the school learners are summarised in Table 1. A total sample of 403 learners was obtained from the three selected high schools. The study entailed 185 (46%) boys and 218 (54%) girls, with a mean age of 16 years (SD = ±2), ranging from 10 to 21 years. Two age groups were created; 10–14 years [n = 100 (25%)] and 15–21 years [n = 303 (75%)]. Learners were distributed across the school grades 8 to 12 and characterised by repeating a grade (33%) and missing school at least three times in a quarter of a year, mainly due alcohol hangover (21%) and illnesses (70%). Most learners walked (61%) to school and were receiving pocket money of at least ZAR 100/week (83%). Alcohol use among learners was estimated at 48%, with beer/cider (55%) and wine (27%) being commonly consumed alcohol types. Approximately 18% of learners were smoking, and among the smokers, cannabis was commonly use (62%) compared to cigarette (38%). The demographic differences between boys and girls were significant for age (*p* = 0.003), school grade (*p* = 0.048), repeated grade (*p* < 0.001), transport mode to school (*p* = 0.006) and receiving pocket money (*p* = 0.038). 

**Figure 1:**
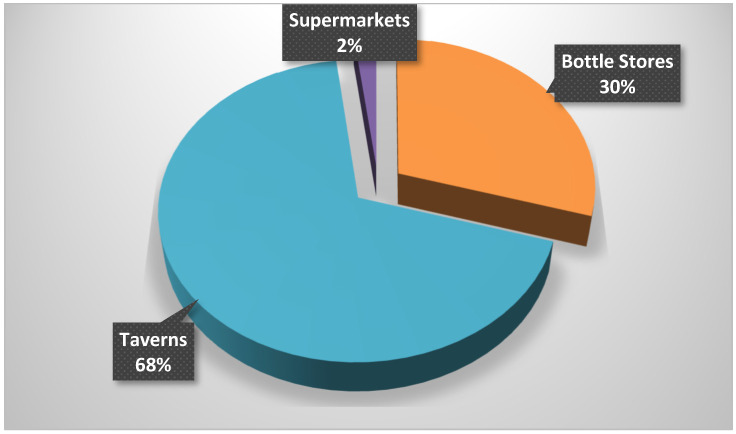
Alcohol outlets for accessibility.

In Figure 1, most learners (68%) reported that they purchase alcohol at the taverns, while 30% at bottle stores and 2% at the supermarkets.

Table 2 shows the accessibility and motivations of alcohol use among school learners, compared using a Chi-squared/Fischer’s exact test. Some of the learners (70%) reported that it is wrong to drink alcohol. Over half of the learners (54%) reported that there was a liquor store near their schools, while 70% indicated that they are able to purchase alcohol without using an identity document. Most learners consumed alcohol at parties (41%), a friend’s place (22%) and clubs (20%). The last places where learners drank alcohol prior to the study were in the community (40%), at home (25%) and at a social/traditional gathering (25%). Almost half of the learners (48%) introduced themselves to alcohol use, influences of friends (36%) and family (14%). Two-thirds of the learners (70%) reported having friends who consume alcohol, while 79% reported that their family members were drinking alcohol regularly, including siblings (26%) and extended family (42%). 

In Table 3, the motivations for alcohol use are reported and compared by sex using Chi-square/Fischer’s exact tests. School learners reported that the main motivation for alcohol use was self-pleasure (36%), coping with stress (23%) and increasing their self-esteem (19%), while other learners said it is good for health (10%). Very few indicated that they drink alcohol for fun (8%), or it helps them to function (3%), with insignificant differences by sex for the trend (*p* = 0.278) and between categories. 

Table 4 shows the effects of alcohol use among school learners compared by sex using Chi-square and Fischer’s exact tests. A total of 83% of the learners reported to have knowledge on the effects of alcohol. Most learners believed that alcohol has a negative impact on health (56%), while 25% reported the effect on brain function, 12% on school work and few felt that there is not much effect (7%). Feelings of sadness (45%) and embarrassment (40%) were reported among learners, especially after drinking alcohol. Alcohol-related social harms affecting learners ranged from problems with friends (25%) and parents (17%), physical fights (19%), risky sexual behaviour (11%), injuries/accidents (10%), violence (10%) and engaging in other substance abuse (7%), with insignificant differences by sex for the trend (*p* = 0.319) and between categories. 

## 4. Discussion

We investigated the accessibility, motivations and effects of alcohol use among high school learners. Poor demographic and lifestyle characteristics were observed among learners in terms of alcohol and tobacco use at an early age. These included having relationships with older partners, walking a distance to school, having jobs during spare time, repeating grades and missing school mainly due alcohol hangover and illnesses. Peri-urban areas, also called townships in South Africa, are known for several challenges in terms of lack of social and economic facilities required to build sustainable communities [53], with low socioeconomic status [17]. Both poor socioeconomic status and infrastructure have been associated with increased alcohol use, promoted by accessibility and availability of alcohol products in the neighbourhood [56,57,58]. This is in addition to poverty and social inequality prevalent in South Africa and implicated in alcohol use, especially heavy drinking [31]. The wide availability of alcohol has been related to poor education performance and higher rates of school absenteeism among young people [41]. 

Accessibility to alcohol outlets close to schools was reported by learners in this study. Further results showed that learners were purchasing alcohol from taverns and bottle stores, and two-thirds reported purchasing without an identity document required. Despite the fact that most of the learners reported that alcohol use is wrong, almost half of them were using alcohol. Learners also consumed alcohol at parties, friend’s places, clubs and communities. These findings confirm ease of alcohol access by learners both around schools and in the communities, consistent with studies conducted in South Africa [21,24,31] and other countries [39,59,60]. Hasheena et al. [28], reported that 62% of grade 8 learners, 81% of grade 10 learners and 92% of grade 12 learners reported that alcohol was easily accessible to them in Namibia. Studies have reported that the more alcohol outlets within school vicinity and in the communities, the more likely youths will engage in drinking alcohol, and this has been reported in countries such as South Africa [21,24,31], Namibia [39], Brazil [59] and Canada [60]. Therefore, the wide availability of alcohol outlets around schools and the lack of regulating policies contribute to the normalisation of alcohol use [60,61,62,63]. In the South African context, the National Liquor Policy prohibits alcohol outlets operating within a 500 m radius to schools and other public places, such as churches, suggesting alcohol outlets are violating the Liquor Policy [23]. Recent literature suggests that South Africa does not have national restrictions on the density of alcohol outlets or the days, hours and location of their operation as of mid-2018 [64]. The more that alcohol outlets in the community operate without age restriction entry, the younger the people who are exposed to drinking alcohol, and that has fatal health outcomes. 

Motivations for alcohol use among learners in the current study were self-pleasure, increasing self-esteem and coping with stress. Abbey et al. [65] have reported that a belief that alcohol helps with coping or social skills puts individuals at risk for abusing alcohol, especially when the appropriate environmental circumstances arise. Studies show that people who reported a higher motivation to drink alcohol in order to cope with negative effects, tend to drink more alcohol, yet encounter more negative alcohol-related consequences [65,66,67]. Most learners, also indicated that after consuming alcohol, they felt sad and embarrassed. Additional studies have found that students reported sadness, fear and shyness after alcohol use [68]. It seems as if alcohol use in the current study was motivated by learners’ feelings, and seeking coping mechanisms, without considering the emotional impact that accompanies alcohol use. Sadness was the most identified feeling among learners in this study, especially after drinking alcohol, which is similar to other researchers’ suggestion [69]. 

Most of the learners in this study indicated that they introduced themselves to alcohol use, although friends and families were also implicated. The current findings correspond with the findings from other studies in South Africa [17,37] that showed that parents who use alcohol have great influence on their adolescents’ drinking behaviour, and the curiosity of experimenting with alcohol use is peer related [70,71]. Further literature documents that some of the strongest influences on adolescent drinking behaviour come from the people that adolescents spend most time with, such as family and friends [38,72,73]. Although the influence of parents on alcohol use among their children diminished with time, parents seem to exert a greater influence among their children before they reach the age of 15 [74]. Social learning has been implicated as an influence on adolescent alcohol use based on the behaviour of other significant persons such as family members and friends, as well as social imitation and social modelling from peers. as reported by Bențea [36]. Parents’ motivation, norms, beliefs, values and goals are imperative if they are to modify behaviour in their adolescent children. Unquestionably, people shift from the social context of the family unit during childhood to focus more on their peers and their schools during adolescence, which might explain the situation in the current study. Nonetheless, parents must promote, monitor and manage the behaviour of adolescents. 

Knowing the effects of alcohol use was common among the learners in the current study. They reported that alcohol use has a negative impact on health, brain function and school work. The alcohol harm paradox suggests that youth living in lower income areas are more exposed to alcohol and suffer more alcohol-related harms than their peers living in less deprived neighbourhoods [61,62]. Early initiation of alcohol use is well documented to lead to alcohol use disorder in adolescence and later in adulthood [41,59]. Behavioural research has found that educational performance is highly correlated with substance abuse [73], and a high level of drinking results in self-reported academic difficulty among high school learners [74]. Additionally, alcohol has toxic effects predisposing users to chronic diseases [26,33,75], especially later in adulthood [41,42]. Learners further reported that in the past 12 months, due to alcohol use, they encountered social harms with friends and parents, had physical fights, injury/accidents, engaged in unprotected sex, had trouble with police and engaged in other substance abuse. Research on alcohol use and delinquent behaviour among adolescents in South Africa has reported that 12.4% were found carrying a handgun in the previous year, while 22.4% had attacked someone with the intention of inflicting serious harm, and 41.3% were involved in serious fights at school [71]. Further, Windle and Zucker [76] have reported that adolescents who regularly consume alcohol are more likely to indulge in other risky behaviours, including smoking, recreational drug use and risky sexual behaviour. Other researchers in South Africa have associated harmful alcohol use with increased incidence of risky sexual risk behaviour and consequently an increased HIV risk in youth [46], negatively impacting the public health in South Africa. 

## 5. Limitations

In terms of limitations, the study may not necessarily represent the entire population of adolescent learners, since only black school learners from the peri-urban high schools participated in the study. This has implications for the generalisability of the findings to other races and settings. Causality could not be concluded from the results due to the cross-sectional and descriptive nature of the study, respectively. Relying exclusively on adolescent self-reports, measures of alcohol consumption might be affected by social desirability, even if anonymity and confidentiality were assured. Lastly, we did not measure biochemical variables that confirm alcohol use, such as plasma activities of gamma-glutamyl transpeptidase, aspartate aminotransferase and the erythrocyte mean corpuscular volume. Future research should endeavour to use the Alcohol Use Disorders Identification Test to screen for alcohol use and study plasma activities of the abovementioned variables and several alcohol-related clinical outcomes. The conclusion of this study could only provide a starting point for other long-term prospective research focusing on identifying and understanding the ease of access to alcohol outlets with motivations and social harms, which might explain alcohol use through the phases of adolescence. 

## 6. Conclusions

The availability of alcohol outlets (i.e., taverns and bottle stores) around schools and easy access to alcohol without enforcing age restrictions support adolescent alcohol use even though the National Liquor Act in South Africa prohibits alcohol sales to individuals younger than 18. There is a need to prioritize limiting adolescent alcohol access and enforcing the South African National Liquor Act. Although alcohol-related problems in school adolescents have been attributed to urban environments, this study confirms that peri-urban settings (i.e., townships) have as much influence. Continuous research on neighbourhood characteristics of alcohol use among school age adolescents is useful for planning public health programs. Additionally, life skills training to address learners’ drinking motives and constantly alerting parents about the relevance of modelling behaviour should be emphasised.

## Figures and Tables

**Table 1:** Demographic characteristics of school learners.

Variables	All	Boys	Girls	*p*-Value
	n (%)	n (%)	n (%)	
Age (years)				
10–14	100 (25)	33 (18)	67 (31)	0.003 *
≥15	303 (75)	152 (82)	151 (69)	
School grade				
8	92 (23)	31 (17)	61 (28)	0.048 *
9	90 (22)	41 (22)	49 (22)	
10	57 (14)	32 (17)	25 (11)	
11	91 (23)	42 (23)	49 (23)	
12	73 (18)	39 (21)	34 (16)	
Repeated grade				
No	272 (67)	104 (56)	168 (77)	<0.001 *
Yes	131 (33)	81 (44)	50 (23)	
Missing class in a quarter				
No	330 (82)	149 (81)	181 (83)	0.518
Yes	73 (18)	36 (19)	37 (17)	
Reasons for missed class (n = 73)				
Hangover	15 (21)	6 (24)	9 (19)	0.673 ”
Illnesses	51 (70)	16 (64)	35 (73)	
Other	7 (9)	3 (12)	4 (8)	
In a relationship				
No	135 (33)	58 (31)	77 (35)	0.400
Yes	268 (67)	127 (69)	141 (65)	
Partner older (n = 268)				
No	146 (54)	66 (54)	80 (55)	0.804
Yes	122 (46)	57 (46)	65 (45)	
Partner ages (years, n = 122)				
15–19	71 (58)	31 (54)	40 (62)	0.424
20–29	51 (42)	26 (46)	25 (38)	
Transport mode to school				
Walk distance	249 (61)	128 (69)	121 (56)	0.006 *”
Bus	71 (18)	25 (14)	46 (21)	
Taxi	77 (19)	32 (17)	45 (20)	
Parents’ car	6 (2)	0	6 (3)	
Receiving pocket money/week				
No	68 (17)	39 (21)	29 (13)	0.038 *
Yes	335 (83)	146 (79)	189 (87)	
Spare time job				
No	365 (91)	162 (88)	203 (93)	0.057
Yes	38 (9)	23 (12)	15 (7)	
Dwelling place				
Brick house	284 (70)	136 (73)	148 (68)	0.454
RDP house	72 (18)	29 (16)	43 (20)	
Shack	47 (12)	20 (11)	27 (12)	
Refrigerator use				
No	17 (4)	10 (5)	7 (3) ^‡^	0.279
Yes	385 (96)	175 (95)	210 (97)	
Religion				
Non-Christian	305 (76)	53 (29)	45 (21)	0.062
Christian	68 (24)	132 (71)	173 (79)	
Current alcohol use				
No	210 (52)	101 (55)	109 (50)	0.358
Yes	193 (48)	84 (45)	109 (50)	
Types of alcohol (n = 193)				
Beer/cider	106 (55)	56 (55)	50 (55)	0.363 ”
Wine	53 (27)	25 (25)	28 (31)	
Spirits	19 (10)	10 (10)	9 (10)	
Traditional beer	15 (8)	11 (10)	4 (4)	
Smoking				
No	160 (82)	86 (92)	74 (80)	0.478
Yes	34 (18)	16 (8)	18 (20)	
Tobacco types (n = 34)				
Cigarette	13 (38)	6 (38)	7 (39)	0.934
Cannabis	21 (62)	10 (62)	11 (61)	

* indicates significant difference (*p* < 0.05); ” indicates Fischer’s exact test used for variables with expected values less than five in a cell; ^‡^ indicates n = 217 for girls, and RDP stands for Reconstruction Development Programme.

**Table 2:** Accessibility and motivations of alcohol use among school learners.

Alcohol Access and Use	All	Boys	Girls	*p*-Value
	n (%)	n (%)	n (%)	
Is it wrong for learners to drink alcohol?				
No	60 (15)	28 (15)	32 (15)	0.855
Not sure	61 (15)	26 (14)	35 (16)	
Yes	282 (70)	131 (71)	151 (69)	
Liquor store near the schools				
No	185 (46)	94 (51)	91 (42)	0.069
Yes	218 (54)	91 (49)	127 (58)	
Identity document required				
No	135 (70)	73 (72)	62 (68)	0.600
Yes	17 (9)	7 (7)	10 (11)	
Sometimes	41 (21)	22 (21)	19 (21)	
Places that learners drink alcohol at (n = 193)				
Party	78 (41)	34 (33)	44 (48)	0.008 *”
Friend’s place	43 (22)	26 (25)	17 (19)	
Clubs	38 (20)	25 (25)	13 (14)	
Bottle store	18 (9)	5 (5)	13 (14)	
Traditional rituals	16 (8)	12 (12)	4 (8)	
Last place drank alcohol (n = 193)				
Community site	76 (40)	44 (43)	32 (36)	0.501 ”
Home site	49 (25)	26 (25)	23 (25)	
Social/traditional gatherings	49 (25)	25 (25)	24 (26)	
I do not remember the site	18 (9)	7 (7)	11 (12)	
School site	1 (1)	0	1 (1)	
Who introduced you to alcohol (n = 193)				
Self	94 (48)	49 (48)	45 (50)	0.420 ”
Friend	69 (36)	34 (33)	35 (38)	
Family	27 (14)	18 (18)	9 (10)	
Neighbour	3 (2)	1 (1)	2 (2)	
Do you have friends who drink alcohol?				
No	119 (30)	58 (31)	61 (28)	0.460
Yes	284 (70)	128 (69)	157 (72)	
Do you have family member who drink alcohol?				
No	86 (21)	45 (24)	41 (19)	0.178
Yes	317 (79)	140 (76)	177 (81)	
Family member drinking alcohol				
Father	60 (19)	30 (21)	30 (17)	0.250
Mother	15 (5)	9 (6)	6 (4)	
Both parents	29 (9)	16 (12)	13 (7)	
Siblings	81 (26)	33 (24)	48 (27)	
Extended Family	132 (42)	52 (37)	80 (45)	

* indicates significant difference (*p* < 0.05); and ” indicates Fischer’s exact test used for variables with expected values less than five in a cell.

**Table 3:** Motivations for alcohol use among the school learners, n = 194.

Proportions of Alcohol Use and Reasons	All N = 403 n (%)	Boys N = 185 n (%)	Girls N = 218 n (%)	*p* Value
Non alcohol users				0.358
No	210 (52)	101(55)	109 (50)	0.358
Yes	193 (48)	84 (45)	109 (50)	
Total	403 (100)	185 (100)	218 (100)	
Alcohol use reasons (n = 193)				0.278 ”
Self-pleasure	70 (36)	35 (34)	35 (38)	0.369
It helps me to cope with stress	46 (24)	25 (24)	21 (23)	0.363
It increases my self esteem	38 (19)	20 (20)	18 (20)	0.450
It is good for my health	19 (10)	12 (12)	7 (8)	0.643
I drink alcohol for fun	15 (8)	8 (8)	7 (8)	0.602
It helps me to function	5 (3)	2 (2)	3 (3)	1.000 ”
Total	193 (100)	102 (100)	91 (100)	

” indicates Fischer’s exact test used for variables with expected values less than five in a cell.

**Table 4:** Effects of alcohol use among school learners.

Effects of Drinking Alcohol	All N = 403 n (%)	Boys N = 185 n (%)	Girls N = 218 n (%)	*p*-Value
Knowledge of the effects of alcohol?				0.378
No	69 (17)	35 (19)	34 (16)	
Yes	334 (83)	150 (81)	184 (84)	
Effects of alcohol use				0.143
Negatively impact health	186 (56)	77 (52)	109 (60)	
Brain functioning	84 (25)	36 (24)	48 (26)	
School work	40 (12)	23 (15)	17 (9)	
Has no effect	22 (7)	13 (9)	9 (5)	
Feeling after drinking alcohol				0.079
Sad	59 (45)	19 (35)	40 (52)	
Embarrassed	53 (40)	28 (52)	25 (32)	
Just fine	19 (15)	7 (13)	12 (16)	
Alcohol related harm in the past 12 months (n = 193)				0.319
Problem with friends	48 (25)	27 (26)	21 (23)	0.586
Physical fight	37 (19)	16 (15)	21 (23)	0.193
Problem with parents	33 (17)	13 (13)	20 (22)	0.089
Engaging in unprotected sex	21 (11)	12 (13)	9 (11)	0.651
Injury/accident	20 (11)	14 (13)	6 (7)	0.189
Trouble with police officers	20 (10)	12 (12)	8 (8)	0.499
Engaged in other substances	14 (7)	8 (8)	6 (6)	0.738
Total	193 (100)	102 (100)	91 (100)	

## Data Availability

The dataset for the school-age adolescents generated and analysed during the current study is available from the corresponding author upon reasonable request.
